# Epigenetic Mechanisms in Latent Epstein-Barr Virus Infection and Associated Cancers

**DOI:** 10.3390/cancers16050991

**Published:** 2024-02-29

**Authors:** Atharva S. Torne, Erle S. Robertson

**Affiliations:** Tumor Virology Program, Department of Otorhinolaryngology-Head and Neck Surgery, Perelman School of Medicine, University of Pennsylvania, Philadelphia, PA 19104, USA; atharva.torne@pennmedicine.upenn.edu

**Keywords:** Epstein–Barr virus, tumor viruses, viral epigenetics, host virus interactions, microRNAs, EBV-associated cancers

## Abstract

**Simple Summary:**

Epstein–Barr virus (EBV) was first isolated in 1964 and has since become an important human tumor virus. With an estimated 90% of the human population infected with the virus, EBV has also been shown to cause several types of cancers. The virus has evolved numerous epigenetic mechanisms by which it can affect its host and contribute to the development and progression of cancer. In this review, we introduce four prominent epigenetic regulatory mechanisms that result in host-virus interactions for the purpose of EBV infection, persistence, and contribution to EBV-associated diseases. We then look at how epigenetic profiles of the host are altered in EBV-associated cancers to understand the precise ways EBV interacts with its host to cause disease. This work explores the viral epigenetics of EBV and provides insights into the knowns and unknowns of research into EBV and EBV-associated cancers.

**Abstract:**

The Epstein–Barr Virus (EBV) is a double-stranded DNA-based human tumor virus that was first isolated in 1964 from lymphoma biopsies. Since its initial discovery, EBV has been identified as a major contributor to numerous cancers and chronic autoimmune disorders. The virus is particularly efficient at infecting B-cells but can also infect epithelial cells, utilizing an array of epigenetic strategies to establish long-term latent infection. The association with histone modifications, alteration of DNA methylation patterns in host and viral genomes, and microRNA targeting of host cell factors are core epigenetic strategies that drive interactions between host and virus, which are necessary for viral persistence and progression of EBV-associated diseases. Therefore, understanding epigenetic regulation and its role in post-entry viral dynamics is an elusive area of EBV research. Here, we present current outlooks of EBV epigenetic regulation as it pertains to viral interactions with its host during latent infection and its propensity to induce tumorigenesis. We review the important epigenetic regulators of EBV latency and explore how the strategies involved during latent infection drive differential epigenetic profiles and host-virus interactions in EBV-associated cancers.

## 1. Introduction

An infectious entity (initially termed a ‘filterable agent’) capable of inducing cancer was first described by Peyton Rous in chicken sarcoma models in 1912 [1,2]. Over thirty years later, Denis Burkitt described a cancer-causing agent of infectious etiology in the 1940s following his observations of extranodal lymphomas in children of sub-Saharan countries referred to as the malarial belt [3]. However, the discovery of the first human tumor virus did not materialize until 1964, when investigators Anthony Epstein, Yvonne Barr, and colleagues isolated a herpesvirus of previously unknown origin from Burkitt lymphoma lymphoblasts, which was provided by Denis Burkitt [4]. Since then, the virus has been referred to as the Epstein–Barr virus (EBV) and later human herpesvirus 4 (HHV-4) to be in line with the international nomenclature. Since this initial isolation, an additional six oncogenic viruses have been identified [5], including the second human gammaherpesvirus, named Kaposi’s sarcoma herpesvirus (KSHV) and human herpesvirus 8 (HHV-8), which belongs to the Rhadinovirus sub-family of gammaherpesviruses [6].

EBV is a member of the Gammaherpesviridae, Lymphocryptovirus subfamily of the Herpesviridae family [7]. Its genome is composed of 184-kilobase linear double-stranded DNA (dsDNA) made up of approximately 59–60% guanine and cytosine [8,9,10,11]. The genome has evolved numerous distinguishable features, notably the insertion of 0.5 kb direct tandem repeats at the terminals [12,13] and various sequence domains with coding capacity interspersed with repeat elements [13]. The genome encompasses open reading frames (ORFs), which encode approximately 80 proteins and 45 untranslated RNAs that aid the virus in pursuing interactions with the host that promote its persistence in vivo [10,13]. It is hypothesized that the co-evolution of EBV with human hosts over its evolutionary history has made the virus particularly efficient at inducing epigenetic changes in both the viral and host genomes. The epidemiological and clinical importance of this virus is based on the high rates of successful infection, which is estimated to be over 90% of all adults) [8,11,14], and the links to infectious diseases [15], autoimmune disorders [16,17], and numerous cancers [18]. The virus today is at the center of all-encompassing research areas ranging from studying the epigenetics of co-evolved genomes to defining the mechanistic frameworks of EBV-mediated oncogenesis to translational research aimed at developing therapeutics to combat EBV infection.

**Figure 1:**
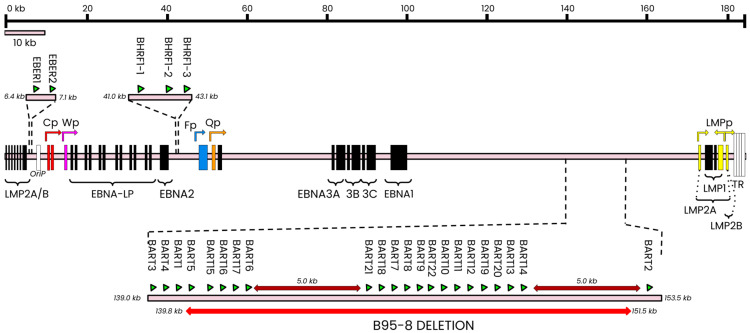
Linearized structure of the wildtype EBV latent genome. The wildtype genome is composed of four latent promoters (Cp, red; Wp, magenta; Qp, orange; LMPp, yellow) that produce mRNA transcripts for the eight major latent antigens. The effect of dysregulated latent promoter activity can sometimes be mitigated by redirecting some latent transcription to certain lytic promoters, such as Fp (light blue) [20]. The major antigens are indicated above as black bars that show the relative position of each antigen’s coding exons. The LMP coding region traverses the terminal repeats and loops back into the ‘start’ of this linear structure. Non-coding RNAs (ncRNAs; termed EBERs in EBV and shown as green triangles) are encoded in a ~700 bp fragment preceding the origin of replication (OriP). Each major latent protein coding region is separated by regions encoding microRNAs (miRNAs); the BamHI rightward fragment 1 (BHRF1) and BamHI rightward A transcript (BART) regions encode twenty-five genomic loci (green triangles; indicative of location only) that produce approximately forty-four mature miRNAs. Spontaneous deletions in the BART region (red underline) are deleted in the B95-8 laboratory strain. Figure adapted from [21].

Epigenetic mechanisms associated with EBV involve a broad range of regulatory machinery that generates non-mutative, heritable, genome-level changes to DNA that can affect both viral and host genomes. From a viral standpoint, reprogramming gene expression during the different latency types and between the latent and lytic stages provides perhaps the most significant evidence for the importance of epigenetic modulation in achieving successful EBV infection. The EBV genome encodes various nuclear antigens (EBNAs), membrane proteins (LMPs), microRNA (ebv-miRs), and non-coding RNAs (ncRNAs; EBERs) [19] (Figure 1), many of whom are expressed only in specific latency types. 

**Figure 2:**
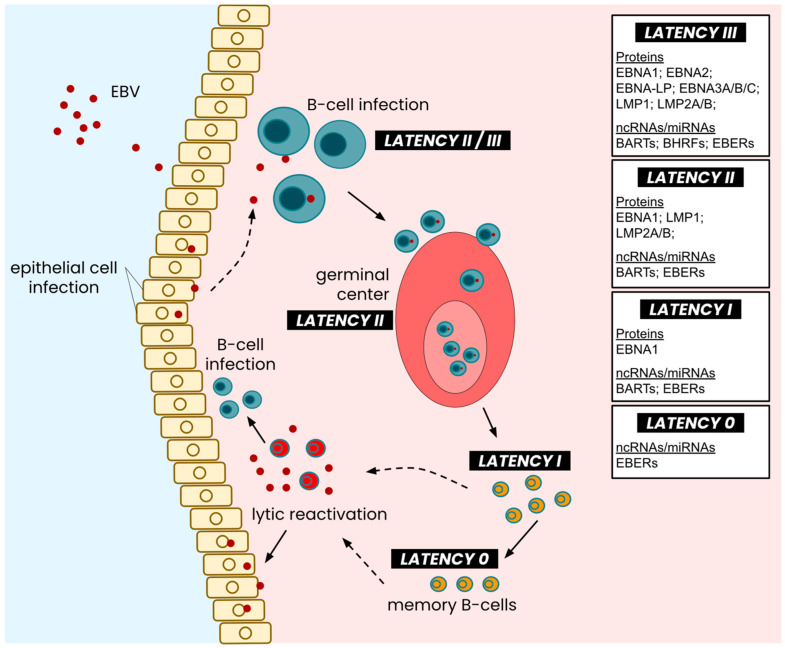
Latency types across the EBV life cycle. Following the entry and infection of host B-cells, EBV undergoes latency III expression, which eventually degrades into latency II expression of its latent genome. As infected cells enter the germinal center and undergo maturation, the EBV genome is suppressed into latency II expression of EBERs, LMPs, and EBNA1, which further devolves into latency I expression as mature B-cells exit the germinal center. Fully mature memory B-cells exhibit a near-quiescent EBV latent genome, where only EBERs are expressed in minimal quantities. Lytic reactivation of the virus enables viral capsid replication and further infection of cells, both lymphocytic and epithelial. Figure adapted from [27].

Upon infection of human B-cells, the virus adopts a latency II genetic program and uses the host DNA polymerase to produce EBNA1 rapidly and any other factors needed to tether the viral episome to host chromosomes [22]. This is followed by type III latency, which encompasses the expression of all latent antigens encoded in the latent genome (Figure 1). The subsequent latent expression generally downgrades into LMP-exclusive type II expression, which continues when B-cells mature in the germinal center. The maturation of B-cells within the germinal center demarcates the end of the active expression of most latent proteins and the adoption of type I latency, where only EBNA1 is expressed [23] (Figure 2). The type II latency program is subcategorized as latency IIa and IIb depending on the expression of LMPs (or, inversely, the expression of EBNAs). For the purpose and context of this review, we look at latency II as a whole. A type 0 latency program has also been described and is primarily considered quiescent and reserved for memory B cells with no viral protein expression, although small non-coding RNAs continue to be expressed within this latency type, much like types I, II, and III [24]. Throughout these latency types, different non-coding RNAs contributing to EBV infection [25,26] are expressed and will be discussed later. While the Cp, Wp, Qp, and LMPp promoters drive latent gene expression, the lytic reactivation of the virus (Figure 2) may sometimes redirect the transcription of some latent antigens to a lytic promoter; however, evidence for the phenomenon remains sparse and conflicting. As such, generally accepted mechanisms involving promoters in the latent and lytic stages continue to indicate latent promoter activity during lytic reactivation, which may even induce the latent promoters further.

The epigenetic mechanisms that drive the activation and repression of viral protein expression contribute to the proliferative behavior of infected B-cells. Generally, the various mRNAs expressed during the different latency programs are responsible for promoting cellular processes that lead to the establishment of latent infection and lifelong persistence of the virus in its hosts (Table 1). In particular, the persistent expression of EBNA1 has been shown to be fundamental for genome replication, acting as a transcriptional activator and modulator, as well as a key replication initiator, which binds to the origin of latent replication referred to as oriP [28]. Further, EBV is also known to usurp numerous host cell factors to modulate viral gene transcription activities to regulate the host gene expression. Hence, the epigenetic potential of EBV makes studying these mechanisms a particularly key area of research, especially as they relate to the continued development of our understanding of host–virus interactions.

Like epigenetic regulation, the epidemiological and clinical significance of EBV cannot be understated. EBV is hypothesized to have successfully infected the vast majority of the global adult population [8,11,14,40,41]. More recently, there is growing evidence that implicates EBV epigenetics as the root cause of or contributor to the development of various autoimmune disorders [16,17] and cancers [18]. Significant immunological evidence points towards a causative role for EBV infection in the development of multiple sclerosis, either through molecular mimicry, enrichment of MS alleles in EBV+ patients, or through epidemiological correlation of EBV antibodies and clinical diagnosis of MS [16]. Furthermore, biopsy data from Burkitt’s lymphomas have confirmed the existence of numerous EBV antigens in the tumor mass, indicating a very high association of the virus with the tumor [42]. Likewise, patients have been shown to possess a higher malignancy risk for carcinoma in a study that linked the presence of high EBV titers to a higher malignancy risk of nasopharyngeal carcinoma [43]. With regards to Hodgkin’s lymphoma (HL), the trend continues, as studies found consistent expression of LMP1 in the histological analysis of HL tumors [44], while another study reported an increased risk for HL in patients with elevated levels of anti-VCA IgG antibodies [45]. As such, studying the virus–host interactions and epigenetic marks that contribute to the propensity to cause disease, as well as identifying putative targets for therapeutic intervention, add to the list of outstanding interests regarding EBV and its role in associated diseases.

The principal aim of this paper is to review our current understanding of epigenetic drivers during EBV latency programs. To that end, we will explore how epigenetic drivers in the EBV genome can promote host–virus interactions and contribute to the long-term persistence of the virus via episome maintenance in the host genome. Subsequently, we will briefly look at how epigenetic imprinting and host–virus interactions are preserved in EBV-associated diseases, focusing specifically on three distinct cancers.

## 2. Epigenetic Regulation in Latent EBV Infection

The lifelong nature of EBV infection has created opportunities to study an array of viral characteristics and functional activities throughout the viral life cycle. Likewise, the distinct phases of the viral lifecycle, like all herpesviruses, perpetrated by complex genetic expression programs, have also contributed to the clear separation in epigenetic changes that EBV exerts on its host. In the latent stage alone, epigenetic regulators have activated major genetic expression programs that allow the immortalization of cells following infection. This section discusses the three key epigenetic regulators associated with EBV latent infection.

### 2.1. DNA Methylation Is a Major Epigenetic Regulator of the EBV Genome

Genetic programming during latent infection is a hallmark of EBV as it establishes successful lifelong persistence in its host. The switch between latent stages (0, I, II, and III) is orchestrated by gene expression and regulation of the BamHI W (Wp), BamHI C (Cp), and BamHI Q (Qp) promoters. While these viral promoters enter infected cells as unmethylated EBV genomic DNA, post-infection DNA methylation of the genome, specifically of these promoters, is critical for the controlled expression of latent proteins dependent on latency type. Wp is particularly prone to DNA methylation [46] and is heavily methylated some days after infection [47]. However, the promoter is never hypermethylated and thus fully silenced; contrary to expectations, it is oftentimes hypomethylated during certain latency types in B-cells to facilitate viral gene expression [47]. Transcriptional repression of the EBV genome, inclusive of the Wp regulatory region, shares a close association, where methylation levels of Wp can directly determine the level of transcriptional repression of viral genes [47]. Host DNMT3b, a DNA methyltransferase, is particularly relevant to Wp regulation as it drives the methylation of sites within the Wp region and aids in the switching between Wp and Cp as EBV switches between latency programs [47]. Although Wp establishes itself as critical during EBV latency by transcribing EBNA2 [48], this very same activity causes it to become optional for all following events due to the EBNA2/EBNA-LP coactivation of Cp [49]. Thus, its overall importance to EBV may be restricted to the naïve EBV genome that is preparing its full-scale gene expression repertoire after establishing infection. The transcriptional activity of Wp is supported by transcription factors CREB [50] and BSAP/Pax5 [51]; however, their respective binding sites contain CpG island sites which, when methylated, block transcription factor binding and thus silence the promoter [47].

The methylation profile of Cp is dynamic and can encompass the two extremities of DNA methylation levels. Cp exists in a hypermethylated state with minimal activity or functional role in type I latency, as evidenced by a lack of mRNA transcripts known to be under Cp transcriptional control [52,53]. However, the necessity to express the full suite of latent genes in type III latency results in the demethylation of Cp to facilitate the full repertoire of EBNA gene expression [53,54]. The two extremes of methylation patterns seen during initial infection are normalized to a moderately methylated Cp on viral episomes belonging to the long-term persistence reservoir. This indicates that methylation is critical to the establishment of infection to promote long-term persistence via gene expression at the minimum necessary level (latency) and elimination of unnecessary biological footprints via acute epigenetic silencing of the viral genome. With regards to the interaction of Cp with host cell factors, Lu and colleagues have demonstrated an interaction between EBNA2 and host TET2 at the RBP-JK sites within the Cp promoter that modulates methylation during the latency III program [55]. Likewise, DNMT3b has again been reported to be significantly upregulated in latency I cells, and it failed to recover Cp activity when it was knocked down in latency III programming, indicating that the potential recruitment of DNMT3b to Cp is likely for the express purpose of maintaining ideal methylation levels that allow expression of the EBNAs [56].

A fundamental feature of Qp is its high methylation potential but an active unmethylated status in vivo [57,58], unseen in neither of the other two promoters discussed above. The Qp promoter has the simplest methylation profile of the three latent EBV promoters, which is indefinitely unmethylated. It is active across all viral gene-expressing latency types (the only exception being latency 0 with only ncRNA expression [24]), is responsible for EBNA1 expression in type I latency [59], and is also autoregulated by the antigen [60]. Contrary to Cp and Wp, the Qp promoter is largely unmethylated and requires physical binding with cellular transcriptional repressor CCCTC-binding factor (CTCF) for protection from DNA methylation. The host CTCF protein has been shown to bind directly to the CpG island upstream of the Qp initiation site, which prevents DNA methylation [20]. The study by Tempera et al. demonstrated the functional importance of the binding site, where site-specific mutations led to significant accumulation of DNA methylation on this promoter and the redirection of gene expression to the lytic Fp promoter [20]. Beyond relying on direct host–virus interactions to modulate its genome, the inherent silencing of Qp in latency II or III highlights a likely function of Qp as a single-source transcription regulator of EBNA1 expression, which is required for latent viral persistence. It is also possible that modulation of Qp transcriptional activities by the host Sp1 transcription factor may further contribute to the regulation of Qp activity [61]. However, the minimal activity of Qp and a lack of Qp-specific viral products not under the control of other promoters make its study particularly challenging.

The dynamic methylation status of EBV genomes during different subtypes of latency has been the subject of various hypotheses. In a 1999 paper by Paulson and Speck, the authors suggested that discrete methylation of EBV promoters directly correlates with EBV’s ability to switch from latency, which is capable of driving immortalization, to the more restricted subtype of latency. This can also facilitate evasion from host immune responses against EBV antigens in immortalized cells and ensure unrestricted transcription of EBNA1 to facilitate propagation or the viral genome [58]. This hypothesis has definitive merit, and the behavior of viral components strongly supports it. The methylation of DNA for switching latency types (as a direct result of promoter switching) is characterized by repression of all latent genes except those necessary to sustain the transitioned latency phase of the virus, which is indicative of activities of the virus to minimize its footprint in the host and thus reduce the likelihood of detection by host immunosurveillance systems. Likewise, the same switch from latency III to I is likely indicative of a switch in viral strategy from active infection to a more dormant program, which is critical for long-term persistence in cells. Without the necessity to express proteins that promote cellular transformation or pro-viral cellular processes, methylation of viral DNA to restrict its transcription is a definitive way to signal the transition from active, immortalizing latency to a restricted latency program such as latency I or 0 (a non-protein-expressing latency comprising only small non-coding viral RNAs). Finally, studies have suggested that EBNA1, the only antigen required for genomic propagation, is under the transcriptional control of multiple EBV promoters [57,62,63]. Using DNA methylation as an epigenetic mechanism to control EBNA1 promoter switching and to ensure that it largely remains under the influence of a more restricted promoter such as Qp suggests an innate tendency of infectious biological systems to minimize their biological footprint upon infection to evade immunosurveillance while also resorting to the most efficient allocation of resources to facilitate persistence. It is important to note, however, that all three promoters contribute to EBNA1 expression, albeit in varying degrees of influence based on mRNA transcript analyses [63]. While that would suggest a form of biological redundancy in latency, it does not negate the fact that EBNA1 is still largely expressed through the restricted Q promoter. As such, studying the mechanism of EBV latency switching presents an opportunity to study the molecular framework within which EBV engages in promoter usage. In addition, identifying whether there are intrinsic host factors, viral factors, or a combination of the two dictates the usage of specific promoters and how these interactions, if any, can affect latency switching.

### 2.2. EBV Modulates Genome Accessibility by Associating with Histone Modifications

Chromatin is a DNA-protein complex incorporating genomic DNA wrapped around globular histone proteins. In addition to their role in condensing DNA, histone amino-tails are free-floating protein terminals amenable to posttranslational modifications that allow chromatin regulation and transcriptional access to genomic DNA (reviewed in [64,65,66,67]). In vitro studies have shown that following B-cell infection, EBV acquires histones to form the nucleosome that is characteristic of genomic DNA in the nucleus. However, patterns of histone modifications are expressly interrupted by latent EBV infection and are a hallmark of various EBV-associated cancers [68,69,70]. However, how EBV succeeds in acquiring and modifying these histones remains unknown.

No unified theory yet has defined the underlying mechanism of histone acquisition by the extrachromosomal EBV genome. The most plausible hypothesis suggests that latent expression of EBNA2 immediately after infection triggers widespread host gene upregulation through dysregulation of RBP-Jκ and causes the dissociation of nucleosomes into free histone pools in the cell [71]. Further, the intrinsic function of histones is to compact DNA; their histone recruitment to free viral DNA will lead to the formation of a nucleosome with the viral genome supercoiled around the histones. Caruso et al. offer some evidence in this regard with their studies of the nuclear lamina following infection [72]. The nuclear lamina is fundamental for regulating chromatin composition. Observations of upregulated lamin A/C correlating with EBV infection suggest that EBV antigens can alter host gene expression to free histones for episomal binding and mimic host DNA for access and integration into the chromosome [72]. The publication also provides evidence for the existence of viral lamin-associated domains (LADs), which allow EBV DNA to localize to the laminar periphery and bind to host lamin A/C [72]. As such, it is possible that the naïve viral genome uses a combination of genome-encoded elements and host cell factors to transition from naïve DNA to a mature DNA-protein complex. The idea also contributes to the notion of viral coevolution with its host, as the incorporation of specific binding domains in the viral genome suggests an intrinsic evolutionary benefit for said interaction associated with successful long-term viral persistence.

At the molecular level, genomic association with histones is principally demarcated with the pairing of genomic elements to specific amino-tail modifications. In a study dedicated to creating an atlas of the EBV epigenome, mapping the EBV epigenome has shown the existence of distinct domains of biological activity and inactivity that were associated with specific marks of histone modification [73]. Transcriptionally inactive and repressed viral promoters are generally associated with heterochromatin marked with repressive marks such as hypermethylation of lysine residues on histone 3 [74]. Conversely, active promoters tend to avoid methylated histones and instead have an affinity for acetylated histones, particularly histone 3 with lysine that is acetylated at positions 4 (H3K4ac), 9 (H3K9ac), and 27 (H3K27ac) [20,73]. The latter is of special importance as H3K27ac may replace the repressive H3K27me3 mark, which has been very sparsely reported on EBV genomes [75]. However, switching latency types (especially between types I and III) also brings about fundamental changes in the pattern of histone modifications associated with latent EBV infection. These patterns are generally affiliated with the active promoter(s) of each latency type; Cp, for example, is heavily enriched with acetylated histones in type III latency, while the same epigenetic modification is absent at Cp in type I latency program [76]. Similarly, epigenetic modifications affiliated with the Qp promoter constitute a mix of acetylated and methylated histones in type I latency; it has no such pattern in latency III and is severely repressed [20,77]. At the whole genome level, latency III generally shows lower levels of association with any histone modifications, as the necessity to express the full latent EBV genome requires significant transcriptional access, which may be limited or disrupted by histones or their amino-tail modifications. This was demonstrated when methylation levels of H3K9me3 were investigated and showed that it is at its lowest during latency type III and at its highest during latency I program [73,77], directly correlating methylation status with promoter activity and the need to activate or repress the full EBV genome.

### 2.3. Chromatin Remodeling of Latent EBV Affects Viral Promoter Activity and Limits the Spread of Histone Modifications across the Genome

Mechanisms discussed thus far highlight the interactions between viral and host factors that aid the viral epigenome in establishing long-term persistence. Conversely, viral remodeling of host chromatin has also been suggested as an epigenetic mechanism through which EBV promotes latent persistence. As discussed above, studies have demonstrated the existence of specific patterns in the deposition of histone modifications that can vary by EBV latency type [74]. Beyond the intrinsic association of the viral epigenome with modified histones, experimental evidence highlights CTCF as a critical factor that binds insulator elements on DNA and aids in the formation of tertiary chromatin loops that facilitate controlled access to the EBV genome and limit the spread of histone modifications [78]. As stated earlier, the activity of CTCF allows Qp activity despite the existence of methylated DNA [20]. With respect to Qp, the existence of a proximal CTCF binding site creates a biological barrier (in the form of bound CTCF) that controls the spread of histone modifications that can interrupt viral genetic programming [72]. Further, studies have shown that the CTCF binding site upstream of Qp also enables the formation of chromatin loops with an enhancer of OriP (origin of latent replication) [79], which further restricts the spreading of post-translational modifications across the viral genome. This results in the association of epigenetic marks with euchromatin and heterochromatin in discernible patterns [20]. A similar process also happens with the LMP promoters and a CTCF binding site upstream of Cp [72], further highlighting the importance of CTCF in the physical manifestation of an epigenetic program perpetrated by EBV. As such, CTCF and its binding sites on viral DNA form a critical component of host–virus interactions that result in the physical reorganization of chromatin to facilitate viral gene expression, as well as switching between latency types or between latent and lytic stages of the viral lifecycle.

Binding sites on viral DNA, encoded genomic instructions, and recruitment of host cell factors likely drive the epigenetic portfolio of EBV that allows it to establish lifelong persistence in its hosts. While a combination of posttranslational modifications and DNA-protein interactions drive this to a large extent, emerging research has also shown the involvement of EBV microRNAs in driving the host–virus interactions that facilitate this process.

### 2.4. EBV microRNAs Modulate Host Gene Expression to Promote Viral Persistence

**Figure 3:**
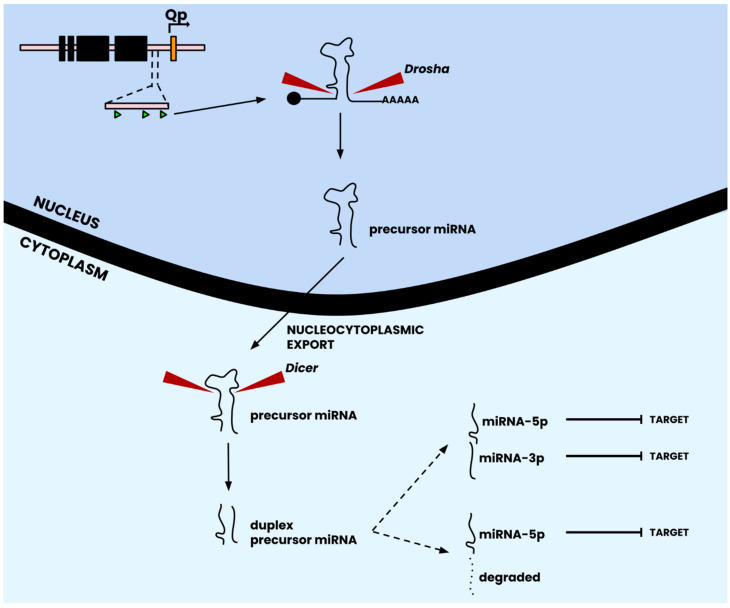
Canonical pathway of miRNA biogenesis. Following transcription from the viral genome, precursor miRNA is processed by the Drosha complex to remove the 5′ cap and poly-A tail to yield a precursor miRNA transcript in its classical stem-loop structure. Following nucleocytoplasmic export, the precursor miRNA transcript is acted upon by the Dicer complex, which removes the stem-loop sequence to yield a duplexed precursor miRNA transcript. The duplex dissociates into two strands, one of which is degraded and one that produces a unique mature miRNA. Depending on the miRNA locus, however, the duplex precursor can also dissociate into two unique, matured miRNAs (which is the case for many EBV miRNAs; see Table 2). Mature miRNAs subsequently are localized to their area of function for targeting. Figure adapted from [82].

Evolutionary principles work towards optimizing our genomes to ensure that they are efficient carriers of genetic information. However, redundancy in biological systems is the norm rather than an exception. Much like genetic redundancy, the epigenetic control infrastructure of EBV has incorporated an RNA-based system for controlling gene expression and maintaining beneficial DNA methylation and histone modification patterns. This system made up of miRNAs no more than twenty-five nucleotides in length, provides a secondary layer of epigenetic control on gene expression by directly binding host and viral mRNA transcripts to promote or repress their translation. According to the canonical pathway of miRNA synthesis, miRNA genes are transcribed into nascent primary miRNAs by RNA polymerases and specific microprocessor complexes [80], and subsequent processing leads to their maturation into miRNAs that can inflict their regulatory effect on host and/or viral factors [81]. An important distinction of the EBV genome in relation to miRNAs is the ability of a small group of genomic loci to produce a much bigger and diverse set of miRNAs (Table 2), which is aided by the processing of duplex precursor sequences by the Dicer complex into two distinct and unique mature miRNAs [81] (Figure 3). Although the canonical pathway suggests that only one strand of the duplex can mature into miRNA, studies have suggested that maturation of both arms of EBV miRNA hairpins into mature miRNAs is possible [82,83] (Figure 3; Table 2).

The EBV genome has two major miRNA encoding regions, BART and BHRF, both of which are flanked by protein-coding regions (Figure 1). The BamHI fragment H rightward open reading frame 1 (BHRF1) cluster encodes BHRF1-1, -2, and -3 miRNAs, while the BamHI fragment A rightward transcript (BART) cluster encodes a group of twenty-two miRNA precursors that produce the 44 currently known miRNAs [86,87] (Figure 1; Table 2). Often, miRNAs are supported in their function by other cellular and/or viral non-coding RNAs (ncRNAs). For this review, we will principally discuss the role of miRNAs in EBV epigenetic control and will cite examples of supporting ncRNAs (cellular or viral) where they form appropriate connections. 

The EBV genome comprises approximately 44 miRNAs produced from 25 precursors (Table 2) that promote epigenetic control of viral gene expression [88,89]. A key feature of these miRNAs is their uracil-rich nature (Table 2), which is also prominent in at least one other virus from the broader Herpesvirus family [90] and also appears to be an evolutionarily important encoded feature of certain small RNAs (sRNAs) of bacterial origin [91]. Substantial research into these miRNAs has been conducted in the context of viral miRNA and host–cell interactions and their contribution to EBV-associated cancers, with the principal roles of viral miRNAs largely responsible for regulating host gene expression [92]. However, miRNA expression does indeed follow some general principles. Like methylation and histone modifications, there is a distinguishable pattern of miRNA expression that directly correlates to the viral latency type. In particular, type III latency is identified by the high levels of BHRF miRNA expression due to their proximity to the Wp and Cp promoters [93], while BART miRNAs are generally expressed during type II latency [85,94] but can also be expressed during the other latency types albeit at varying levels [28,95]. It is important to consider that BART miRNAs only undergo high-level expression during type II latency, and numerous studies have also detected the presence of BART miRNAs in most latency types of infected epithelial and B-cells, albeit with some disagreement on the true quantity of expression [85,94,96,97]. The pattern of expression generally extends to the type of cells EBV infects; epithelial cell lines (which typically exhibit the type II latency program) generally exhibit widespread expression of BART miRNAs, while T-cell lymphomas and infected B-cells in general, are known to have very specific subsets of BART miRNA expression [98], possibly due to large genomic deletions in the miRNA encoding regions [99]. This may be in part due to miRNAs being transcribed from large introns of the genome that encode the miRNA; this is indeed the case for BART miRNAs, which are transcribed from a large intron and are correlated with an accumulation of a specific mRNA splice variant containing specific exon joining [100]. As such, differential expression of the BART/BHRF mRNA may directly affect their encoded miRNA expression. The reliance on other transcripts creates a complex system that relies on all aspects of epigenetic regulation discussed thus far, where the repressive action of DNA methylation and histone methylation limits viral gene expression, which in turn affects miRNA epigenetic regulators that rely on intron transcription for their own expression. Cumulatively, this indicates that not only is viral miRNA expression cell-line dependent, but epigenetic modulation via viral DNA methylation may impart a repressive effect on a secondary epigenetic regulator responsible for the downstream targeting of viral and host factors/mRNA transcripts. The cell line-specific nature of miRNA expression has been shown previously [101]; however, whether these changes are purely due to epigenetic mechanisms or a combination of mechanisms remains to be seen.

Parallel to their role as epigenetic modulators at the viral genome level, EBV-encoded miRNAs target both viral and host mRNA transcripts to directly influence expression. Certain BART miRNAs and miR-17/106/20/93 ncRNAs are known to interact with the 3′UTRs of LMP1, LMP2A, and EBNA2 to downregulate the expression of their protein product [84,102]. While the specific downstream effects of these interactions remain elusive, studies in epithelial cells confirm that miRNA-mediated downregulation of LMP1 affects NF-kB activation and increases cell resistance to apoptotic stimuli [92]. Likewise, interactions between viral miRNAs and host cell transcripts have been shown to affect viral immune evasion, cellular apoptosis, and signal transduction in numerous pathways. Following infection, EBV miR-BART2-5p miRNA blocks the activation of NK cells by binding the NKG2G receptor ligand MICB, which acts in a sensory capacity to seek and eliminate infected cells [103]. Similarly, the miR-BHRF1-3 miRNAs are responsible for inhibiting the activity of the interferon-inducible chemokine CXCL11/I-TAC, which binds T cell chemokine receptors and aids in the selective destruction of EBV-infected cells [92,93]. Xia et al. demonstrated that repression of the miRNA enabled the rescue of CXCL11 levels in cells, which indicated a direct host–virus binding interaction that aided viral persistence.

Changes in host signal transduction are also driven by the network of miRNA and host cell factor interactions. An inhibitory effect of BART miRNAs upon LMP1 expression has been shown to alter the NF-kB signaling pathway [104], which is perpetrated by miR-BART1/miR-BART3 stabilization of IκBα and disruption of NF-kB activation via an as-of-yet unknown mechanism [105]. High throughput studies from Skalsky et al. offer the most comprehensive evidence of interactions between miRNAs and host cell factors as they relate to modulating signal transduction. E3 ligases [106], deubiquitinases [107], and multifunctional ubiquitinases/signaling molecules such as PELI1 [104,108], are all known targets of EBV miRNAs in infected B cells, which can bind one or multiple BART miRNAs [104]. The Wnt signaling pathway also offers compelling evidence for interactions between miRNAs and host cells that may contribute to latent infection. EBV miRNAs target and regulate both activators and inhibitors of Wnt signaling. Chen et al. expanded the scope of miRNA targets in the host, using functional screens to elucidate 54 new miRNA targets associated with signaling pathways and biochemically validating five of them as interacting with various EBV miRNAs through their respective 3′UTRs [109]. Likewise, just five EBV miRNAs were responsible for affecting the sensitivity of B-cells to BCR crosslinking to affect the downstream activation of NF-kB [109].

The effect of miRNA epigenetic control is also particularly observed in the immune responses of cells following infection. BART miRNAs particularly highlight the diversity of host targets in miRNA-affiliated epigenetic control in this regard. Members of the BART miRNA family suppress innate immune response signaling by targeting RIG1, an inducible gene that uses retinoic acid for expression [110]. BART miRNAs further suppress immunity against tumors by targeting STAT1 and IFN-γ to alter transduction in their respective signaling pathways [111], target transcriptional coactivators for inhibiting type I IFN signaling [112], and bind inflammasome proteins to inhibit the production of signaling molecules, IL-1β and IL-18 [113].

In the context of apoptosis, viral BART miRNAs have been shown to interact with host cell factors to protect against apoptotic stimuli. Skalsky et al. provided the most comprehensive evidence for this by demonstrating the existence of nearly 7827 miRNA binding sites on 3492 cellular 3′UTRs [104]. Skalsky et al. also showed that BART miRNAs bound to the 3′UTR region of BCL2L11 to repress anti-apoptotic activity in LCL cells to enhance their survivability [104], which was already suggested to bind EBV miRNAs [114]. Beyond this, miR-BART4-5p was shown to regulate host BID expression to prevent cell apoptosis. BID, alternatively known as the BH3-interacting domain death agonist, is a pro-apoptotic protein known to amplify caspase activation by linking the mitochondrial and death receptor-mediated apoptotic pathways [115]. Similarly, another manuscript showed that miR-BART22 was initially thought to be a direct binding partner of MAP35K and can affect p38/MAPK signaling in cells [116], although later studies contradicted this finding and showed that it may bind to CASP3 instead in a co-binding capacity with other miRNAs [117].

By promoting cell survival (through inhibition of apoptosis) and modulating cell signal transduction and immune responses, the EBV episome successfully establishes itself in the host by maintaining a microenvironment necessary to evade immune detection. Their role in defining and switching between latent genetic programs further demonstrates their importance to EBV latency. Likewise, miRNAs also add a layer of complexity to viral genetics and promote host–virus interactions that, when combined with other epigenetic regulators discussed herein, contribute to tumorigenic [18], autoimmune [16,17], and infection [15] related diseases.

## 3. Host Virus Interactions and Epigenetic Hallmarks in EBV-Associated Tumorigenesis

The epigenetic mechanisms and hallmarks imparted upon the host genome by latent EBV make the virus particularly potent in contributing to the pathologies of numerous diseases. EBV is particularly known as a viral agent contributing to the progression of diseases such as infectious mononucleosis [15], multiple sclerosis [16,17], and a range of different cancers [18]. Within these cancers, EBV-infected cells are driven towards tumorigenesis by the activation of cancer hallmarks as a direct result of host–virus interactions in nasopharyngeal carcinomas (NPCs) [118], Burkitt lymphomas (BL) [69], EBV-associated gastric cancers (EBVaGC) [119], and other associated cancers. Another associated malignancy, termed EBV-associated lymphoproliferative disorder, is a prominent rival cancer associated with EBV and is reviewed extensively elsewhere [120,121,122].

### 3.1. Nasopharyngeal Carcinoma (NPC)

Nasopharyngeal carcinoma is characterized by malignant neoplasmic growth in the nasopharynx tissues. NPC is endemic to North Africa and Southeast Asia and has an occurrence rate of 25–50 cases per 100,000 people, according to epidemiologic estimates [123,124]. NPC can be classified into three main subtypes: keratinizing squamous cell carcinoma, non-keratinizing squamous cell carcinoma, and undifferentiated or poorly differentiated carcinoma [123]. Specific haplotypes of MHC class I molecules indicate a genetic predisposition to the disease in southern Chinese populations [125]. However, the risk of developing cancer is significantly elevated following exposure to certain EBV subtypes [126]. EBV contributes to NPC tumorigenesis by engaging in a complex interplay between host and virus through latent proteins and miRNAs that create global epigenetic changes in the host genome and the activation of numerous hallmarks of cancer development, including genomic instability [127], uncontrolled cell division [128], and resistance to apoptosis [129].

Host virus interactions contributing to the development of NPC are found across the entire spectrum of biological functions in cells. Principally, LMP1 promotes the expression of chemokines by interacting with carboxy terminal activating region (CTAR)1 and CTAR2, which are responsible for regulating the synthesis of macrophage inflammatory proteins (MIPs) MIP1-α and MIP1-β [130]. This is supplemented by non-coding encoded RNAs (EBERs) that regulate the inflammatory response by increasing TNF-α levels in NPCs by modulating the TLR3 pathway [131]. BART miRNAs are also known to inhibit IFN-β responses as a direct result of EBV infection, and the same is true in NPCs [110]. The recruitment of regulatory immune cells is also present in the tumor microenvironment of NPCs, which is indicative of host–virus interactions to prevent immune detection of the virus. Similarly, EBNA1 directs immunosuppressive FoxP3+ regulatory T-lymphocytes (Tregs) towards the tumor microenvironment by stimulating T-lymphocytes to convert into Tregs, which is achieved by direct interactions through the TGF-β signaling pathway [132,133]. A fundamental characteristic of NPC angiogenesis is regulated by EBV infection [128]. EBV upregulates numerous cellular components, including chemokines [134], transcription factor activator proteins [135], endothelial growth factors [135], and vascular cell adhesion molecules [136] through binding or interactive action of EBNA1 and various miRNAs. Likewise, LMP1 and LMP2A significantly alter the VEGF/VEGFR1 and mTOR pathways, respectively, to induce the formation of endothelial cell deficient vasculature by vasculogenic mimicry [137,138], and EBV miRNAs upregulate the expression of checkpoint programmed cell death protein (PD1) [139], which is also acted upon in a modulatory capacity by LMP1 through at least three different signaling pathways [140]. We limit our discussions on these topics here as other reviews [128,141] have discussed the complex interplay between EBV proteins and nasopharyngeal carcinomas in substantial detail.

Epigenetic hallmarks of NPC perpetrated by host–virus interactions can be predominantly found in the form of aberrant methylation and, to a lesser extent, histone mark depositions and the association of viral genome at these marks. Bioinformatics studies have revealed that at least 3000 genomic sites are hypermethylated in EBV-associated NPC, which directly translates to 668 upregulated and 594 downregulated genes in infected host cells [142]. Another study from Tunisian patient samples has shown excessive hypermethylation of RARβ2 promoters [143], which control the methylation-dependent expression of RARβ ligand-activated transcription factor [144]. A study potentially linking chromatin remodeling with EBV in NPC has suggested that EBV proteins alter the three-dimensional conformation of cellular chromatin to limit access to CTCF binding sites, which creates differentially hypo-methylated regions on the genome [145]. Studies comparing NPC and non-NPC tissues from the nasopharynx have highlighted excessive hypermethylation of lysine 27 on histone 3 (H3K27me3), which is directly correlated to tumor metastasis [146]. Finally, H3K27me3 has also been implicated with EBV infection in NPC as a bivalent switch with H3K4me3, which cumulatively provides compelling evidence for the niche epigenetic hallmarks that are associated with NPC and deposited because of EBV infection. The role of miRNAs in depositing epigenetic marks is also an area of research, although current research mostly limits the role of miRNAs in NPC to wider cellular processes that drive the development of NPC [88].

### 3.2. EBV-Associated Gastric Cancers (EBVaGC)

EBV-associated gastric cancers account for anywhere between 1.3% to 30.9% of gastric cancer cases based on geographic location [147] and have a global average of 8.9% of all gastric cancers annually, with a higher prevalence in males [148]. EBVaGC manifests as an ulcerated, saucer-like tumor in patients and has a specific affinity to manifest in the upper to middle portions of the stomach [147]. The association of EBV with this cancer is particularly important, and research has shown that various elements of the viral genome contribute to the promotion of tumorigenesis [147]. A combination of viral proteins and miRNAs, particularly LMPs and BART miRNAs, contribute to the resistance of infected cells to apoptosis, uncontrolled proliferation, and the deposition of epigenetic marks on the genome.

LMP2A offers a good example of host–virus interaction in EBVaGC. LMP2A is hypothesized to serve as a molecular mechanism to repress cell apoptosis in gastric cancer models [149]. Further, there is evidence that confirms LMP2A is present in up to 40% of all gastric cancers [150]. Another study reports the upregulation of survivin in humans by LMP2A, which suggests that LMP2A may be an indirect modulator of survivin expression via the NF-kB pathway [151]. However, due to a lack of convenient experimental models, studies with LMP2A are limited in their scope. There is not much discernible distinction to be made that allows specific host–virus interactions to be considered as those promoting tumorigenesis or promoting the demarcation of epigenetic hallmarks observed in EBVaGC, although research with miRNAs has shown modulation of specific cellular processes that eventually contribute to tumorigenesis. For example, EBV miRNAs have confirmed roles in modulating epithelial-to-mesenchymal transition [152], upregulating E-cadherin expression [153], binding to cell cycle factors [154], inhibiting pro-apoptosis proteins [155], promoting malignant cell transformation [155], and controlling the movement of killer immune cells into the tumor microenvironment by controlling the senescence-altering sensory phenotypes [156]. Independent events within these processes and their modulation by EBV miRNAs can promote tumorigenesis, although further research is needed to understand the extent to which EBV interferes in these processes and promotes tumorigenesis.

Much like other EBV-associated diseases, EBVaGC is particularly noted for presenting an extensively hypermethylated host genome, possibly a result of EBV infection. CpG island methylation is now considered a hallmark of epigenetic abnormalities in cancers, including gastric cancers, and groups studying EBVaGC have shown significantly more frequent CpG island methylation than non-EBVaGC (EBVnGC) [157,158,159,160], indicating that viral components likely interact with the host genome and leave methylation patterns as an epigenetic mark of host–virus interactions. A deep dive into elucidating these interactions has identified specific genes that are hypermethylated in EBVaGC but not in EBVnGC, with three genes, CXXC4, TIMP2, and PLXND1, showing consistent methylation in EBVaGC [161]. Beyond DNA methylation, EBVaGC also has a distinct association of histone modifications with the transcriptional repression of long interspersed element 1 (LINE-1) [162]. A comparative study between EBVaGC and EBVnGC demonstrated a consistent association of lysine 9 trimethylation of the H3 histone (H3K9me3) with the transcriptional repression of LINE-1 in EBVaGC [162]. The existence of host–virus interactions in EBVaGC is an area of significant research interest. While the importance of specific host–virus interactions remains to be elucidated, compelling evidence from EBVaGC epigenetic profiles suggests that the virus induces highly specific changes in the host genome via viral antigen interactions that promote tumorigenesis.

### 3.3. Burkitt Lymphoma (BL)

Burkitt lymphoma is an aggressive B-cell lymphoma with a rapid doubling time and high prevalence in young males [163]. From a genetic standpoint, BL is associated with significant translocation-induced overexpression of the MYC oncogene, which encodes a transcription factor with oversight on cell proliferation and apoptosis, among other cellular processes [164,165]. The overexpression is largely a by-product of chromosomal translocation of the MYC-encoding genomic region between chromosomes 8 and 14 [166,167,168] as the predominant translocation but may also have other translocations. BL can be broadly classified into sporadic, endemic, and immunodeficiency-associated subtypes, of which the endemic variety is almost always associated with EBV persistence in patients [163,169]. Beyond its tumorigenic features, BL is one of few cancers with an extensive history of association with infectious diseases. *Plasmodium falciparum* is now generally considered a cofactor in endemic BL (eBL) that can drive EBV-mediated tumorigenesis by either suppressing the T-/NK-cell immune response to allow EBV-infected B cell proliferation [170], or by the *P. falciparum* exposure-induced increase in the expression of activation-induced cytidine deaminase (AID) [171], the downstream consequence of which may lead to the hallmark chromosomal translocation of *c-myc* seen in eBL. Fundamental interactions between EBV and its host can promote BL tumorigenesis, and epigenetic marks such as DNA methylation patterns that are very apparent in most cases perform critical gene regulatory functions. A fundamental feature of EBV association with the tumor is its ability to control apoptotic tendencies of the cell without any direct genomic mutations. One study has shown that BLs lacking EBV have significantly more mutations in apoptotic genes that can theoretically compensate for the lack of EBV gene expression that usually controls and prevents apoptosis [172].

Unlike NPC and EBVaGC, EBV-associated BL has a particularly close relationship between epigenetic hallmarks and host–virus interactions, which drives the disease state. The EBV episome has a particular affinity for docking to the host genome at sites that are specifically enriched with B-cell factors and repressive histone marks [173]. The affinity for repressive histone marks is particularly important as EBV-associated BL is generally identified by latent EBV genomes that actively avoid immune detection. As such, associating with heterochromatin is especially beneficial as it promotes transcriptional repression. Another study also found that the binding sites of viral episomes generally contain genes for neuronal function that are also enriched for protein kinase A pathway [173], further demonstrating the specificity of episomal binding to sites that are likely to remain repressed in B-cells, as the likelihood of a neuronal protein being fundamental for B-cell function is low. Although this host–virus interaction does not directly utilize any viral epigenetic mechanisms, it is a fundamental interaction that can be considered a precursor to epigenetic hallmarks that are characteristic of Burkitt lymphoma.

While three-dimensional docking interactions between the host and EBV are fundamental, the bulk of the epigenetic hallmarks perpetrated by this docking occur at the global genomic level because of the post-latency gene expression of the virus. Epigenetic marks on host promoters and heterochromatin are generally theorized to be the result of the viral infection in EBV-associated BL. The inhibitor of the DNA-binding 3 (ID3) gene, which encodes a DNA-binding protein inhibitor, is a particularly good example of epigenetic marks on regulatory elements of genes. The ID3 promoter has been shown to be hypermethylated following EBV infection, and the expression of ID3 has been shown to be silenced by the direct effect of an LMP1-mediated molecular mechanism [174]. Most of our knowledge about LMP1-mediated host–virus epigenetic interactions are a direct result of its modulation of the NF-kB signaling pathway [175], and the increased methylation of key host promoters suggests a crucial role for LMP1-mediated NF-kB signaling modulation in viral persistence and tumorigenesis. Likewise, the persistence of EBV in BL (versus non-EBV associated BL) further shows methylation marks on RUNX1 and lysine (K)-specific demethylase 2B (KDM2B), which, although having opposing roles in tumor progression, are significantly methylated in EBV-associated BL when compared to non-EBV-associated BL [174]. Fundamentally, however, an epigenetic hallmark of EBV-associated BL is global hypo-methylation of the host genome with net hypermethylation occurring only at specific sites, notably within CpG islands when compared to germinal center B-cells [176,177]. With regard to chromosome-level epigenetic hallmarks, DNA methylation is particularly unidirectional on heterochromatin and is largely towards hypo-methylation [178]. It is, however, important to note that the net effect of these methylation patterns is still global hypo-methylation of the genome in BL [176]. Most of these epigenetic features are likely imprinted into the genome because of viral modulation of many cellular processes critical for driving the oncogenic process. In particular, the likely modulation of signaling pathways by LMPs and other viral proteins could result in global methylation changes and chromatin association.

**Figure 4:**
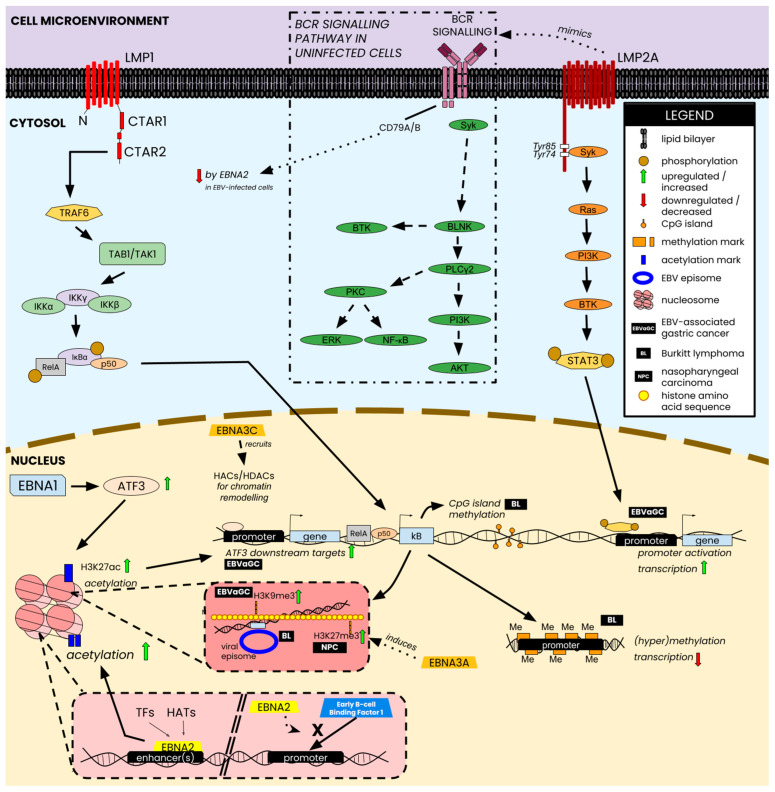
Selected epigenetic changes and host-virus interactions perpetrated by EBV nuclear antigens and latent membrane proteins. Nuclear antigens (e.g., EBNA1) interact directly with transcription factors and/or intermediate proteins of signaling pathways that lead to the deposition of acetylation marks on histones, which translate into the upregulation of downstream targets of the corresponding intermediate, shown in the figure, as ATF3. EBNA2 can also bind DNA sites in the enhancer and promote transcription by attracting histone acetyltransferases (HATs) or transcription factors (TFs) to those sites. Alternatively, EBNA2 can also block the binding of transcription factors to promoters and repress the downstream targets of TFs, such as the EBNA2-mediated repression of CD79B via TF-blocking at the promoter. From a chromatin modeling perspective, EBNA3C recruits histone HATs and histone deacetylases (HDACs) to controlhistone configuration, thereby controlling transcriptional access for both viral and host TFs. Membrane proteins perpetrate similar changes via modulation of signaling activity. LMP1 modulates the NF-kB signaling pathway to inflict epigenetic changes such as CpG island methylation, lysine-specific histone methylation, and promoter-specific methylation to control gene transcription in EBVaGC, BL, and NPC. LMP2A mimics the BCR signaling cascade and directly targets the phosphorylation of STAT3, which subsequently binds and activates key promoters such as those that controls transcription of some factors EBVaGC. Figure composed using information from [28,38,179,180,181,182,183].

A variety of changes occur in the host across the three types of malignancies discussed in this review, ranging from epigenetic mark depositions and differential regulation of host genes to outright alterations to host cell signaling pathways such as the NF-kB pathway. Additionally, the interactions between host factors and various EBNAs—particularly EBNA1, EBNA2, and EBNA3A/B/C—aid in orchestrating some downstream changes associated with the epigenetic changes discussed above. Figure 4 provides a brief overview of a selection of epigenetic changes perpetrated by EBV and how latent antigens contribute to some of the host–virus interaction-induced characteristics of EBV-associated malignancies.

## 4. Future Research Directions

Elucidating the mechanisms by which EBV affects the host genome is a fundamental priority for future directions in EBV research. In particular, the interplay between viral LMP proteins and their role in modulating the NF-kB signaling pathway as well as other signaling pathways, are of particular importance considering their involvement in numerous host–virus interactions. The role of the EBNA proteins and miRNAs is also an area that needs further exploration and will likely provide new information as to their roles in regulating gene expression, cell cycle, and other major cellular activities. Beyond that, three key areas of research need further addressing in the realm of EBV research.

### 4.1. Genetic Evolution of EBV Latent Proteins and Polymerases

EBV proteins have been documented to have geographically significant sequence variation [184]. LMP1 and EBNA1 are two classic examples where sequence variation can be used to study the association of EBV subtypes to diseases [185,186]. However, the lack of comprehensive data for any latent EBV antigen that can accurately predict an EBV subtype, its global epidemiological prevalence, and its propensity to cause disease presents an important challenge for EBV research. Beyond just the clinical and epidemiological significance, further studies into viral genetic evolution can also aid in the development of our understanding of how epigenetic marks are affected in patients with different EBV subtypes. For example, the EBV B98-8 strain has significant deletions in the BART-encoding regions of the genome, indicating that various host–virus interactions and their consequential epigenetic marks never materialize in infected hosts. Even though the B95.8 strain is infectious despite the deletion, how does the absence of a significant set of EBV-encoded miRNAs affect the post-infection dynamics of the virus? BART miRNAs are also implicated in a whole spectrum of biological processes in malignant cells. How does the virus compensate for their suppression while still establishing infection and promoting tumorigenesis? Does the lack of major miRNAs promote infection in one cell type more than the other? One study has suggested that B95.8 viral particles produced in HEK293 (epithelial) cells but present in B cells can re-infect epithelial cells with a higher efficiency [187]. However, the specific molecular mechanisms that enable this enhancement are yet to be understood, as are the epigenetic consequences that arise in the host cells following infection.

### 4.2. EBV in Epithelial Cells

Latent EBV infection in epithelial cells is restricted to type II latency that does not express the full suite of latent proteins [188]. Elucidating the mechanism that controls EBV genetic program restriction in these cells is particularly important in the context of EBV-associated gastric cancers, where EBV has been shown to contribute to tumorigenesis and leave distinct epigenetic marks on the genome [189]. On a similar footing, studying EBV’s association with NPC is equally important in the context of the timeline of the development of tumors. Certain hypotheses suggest that EBV might be establishing aberrant latency in epithelial cells already showing pre-malignant genomic instability [190]. Thus, the question arises: does EBV actively seek and infect these pre-malignant epithelial cells in the nasopharyngeal epithelium during initial infection? Alternatively, is initial EBV infection of epithelial cells random and it is the virions from reactivated B-cells that seek out these pre-malignant epithelial cells? In either case, is there an encoded instruction in the viral genome that contributes to this phenomenon? Likewise, are there any biochemical signals that induce changes in EBV genetic programming that act as a switch to promote tumorigenesis?

If there is no viral persistence in vivo, it is imperative to understand whether undetected neoplasms in uninfected individuals are responsible for producing characteristic biochemical signals, including paracrine activities that attract EBV virions to the nasopharyngeal epithelium when hosts are exposed to the virus. Conversely, if EBV does indeed exist latently in host B-cells, is there any biological incentive for EBV to enter epithelial cells? In the case of nasopharyngeal cancers, it is a sound assumption that increased angiogenesis redirects more nutrients and oxygen to the affected region and thus creates an opportunity for viral proliferation. A localized oxygen concentration increase can induce oxidative stress and recruit immune cells to the affected site, as oxidative stress is also a hallmark of viral infection [191]. However, impairment of the immune response due to neoplasmic modulation of cell signaling could create enough of a mask that detracts cytotoxic T-cell recruitment while promoting conditions where the EBV episome can persist for lytic activation. However, EBV-associated cancers are largely known for being associated with latent EBV [128,192,193], which further brings into question the strict relationship between EBV and its associated cancers. Particularly, is the virus driving the tumorigenic phenotype after establishing latent infection, or is it attracted to existing early-stage malignant cells where it can promote tumorigenesis by modulating host gene expression under the radar of misfiring cell signals? The development of model EBV-positive latent epithelial cell lines can help answer some of these questions. However, they remain difficult due to current limitations in our understanding of why epithelial cells cannot maintain certain EBV latency types during in vitro cell culture, and infection of epithelial cells mostly results in lytic activities.

### 4.3. The Road to EBV Therapeutics

Therapeutic options against EBV are sparse, and most EBV-associated diseases are treated for symptomatic relief or using immunosuppressors and immunomodulators, which have been mostly unsuccessful outside of individual successes [194,195]. Elucidating key viral mechanisms or surface properties have shown promise as they relate to identifying targets for antivirals or vaccines [196], and multiple vaccine types have been preclinically tested [197,198,199,200]. A phase II clinical trial for an adjuvant-based vaccine targeting gp350, the most abundant glycoprotein on the outer viral surface, showed positive data but failed to provide protection against asymptomatic infection, instead only aiding in the prevention of infectious mononucleosis [196]. A fundamental problem with developing any therapeutic option against EBV is the lack of well-defined animal models for studies [196]. EBV’s exclusivity in infecting humans limits the scope of preclinical research into any potential candidates, and well-founded legal and ethical limitations on human testing at the preclinical stage create a bottleneck where promising candidates are abandoned due to insufficiently convincing data in non-human models. As such, the most fundamental research in the realm of EBV therapeutics continues to be the development of an animal model that accurately describes EBV infection as it occurs in humans. Certain avenues, like the humanization of mice, offer some promise [201], and identification of an EBV homolog in rhesus monkeys [202] may eventually offer a comparable model for EBV in nonhuman primates. However, substantial research is needed for either option to become viable for therapeutic and translational research.

## 5. Conclusions

Since its first isolation in 1964 [4], research into EBV has shown the complex interplay between viral and host components that drive its infection and contribute to the pathogenesis of associated diseases. The ubiquitous nature of the virus, likely due to its ease in infecting host cells, has allowed seminal research into infection, proliferation, and egress mechanisms of this virus. As such, EBV is now at the forefront of virological research, especially as it pertains to studying basic viral dynamics and how infectious entities contribute to the development and progression of cancers in humans.

A fundamental feature of EBV is its ability to inflict epigenetic changes in the host genome through host–virus interactions that allow it to evade immune detection and promote tumorigenesis. Four epigenetic drivers, DNA methylation, chromatin remodeling, histone modifications, and EBV miRNAs, aid the virus in establishing infection and are reviewed here. In particular, the role of miRNAs cannot be understated. Despite their comparatively short sequence length, EBV miRNAs control some of the most crucial cellular processes, such as signal transduction and apoptosis, which allows mechanisms such as DNA methylation and chromatin remodeling to operate in the background and exert influence on the host in the form of host gene regulation.

In relation to EBV-associated diseases, the host–virus interactions perpetrated by the three major latent epigenetic regulators affect the epigenetic profiles and progression of three major cancers: NPCs, EBVaGC, and BL, as discussed here. While the association between viral infection and cancers is well-studied, numerous questions regarding basic viral dynamics remain unanswered, which has, to an extent, limited the scope of research into how EBV contributes to these cancers at the molecular level. Prioritization of research on EBV infection of epithelial cells can aid in understanding the dynamics of the virus in a latency-limiting cell line. Likewise, elucidating novel host–virus interactions that promote immunological dysregulation of hosts can aid in the identification of putative targets for antiviral and/or vaccine development, thus reducing the clinical burden of EBV infection and EBV-associated diseases such as infectious mononucleosis, multiple sclerosis, and various cancers.

## Figures and Tables

**Table 1:** Functions of EBV proteins in latent infection.

Protein	Functions	Refs.
EBNA1	Required for efficient viral genome replication and persistence in proliferating infected cells	[29,30]
EBNA-LP	Reduces EBNA2 binding site occupancy by eliminating repressors; coactivator of EBNA2 transcription	[28,31]
EBNA2	Transcriptional activator; deposits H3K4me1 epigenetic marks on histones and depletes nucleosomes	[32,33]
EBNA3A	Engages in polycomb group-mediated epigenetic silencing of CXCL9/10 on host genome	[34]
EBNA3B	Inhibitory role in growth through upregulation of CXCL10 chemokines; putative tumor suppressor	[28]
EBNA3C	Coactivates LMP1 promoter with EBNA2; regulates chromatin remodeling via histone deacetyltransferase recruitment; inhibits apoptosis by modulating IRFs	[28,35,36]
LMP1	Mimics CD40 signaling; activates NF-kB and p38 pathways; essential for EBV-mediated cell transformation	[28,37]
LMP2A	Mimics BCR signaling; promotes growth and cell cycle induction; upregulates IL10 and other anti-apoptotic chemokines and factors in B-cells	[28,38,39]
LMP2B	Negatively regulates the function of LMP2A; lowers BCR crosslinking threshold needed for lytic reactivation	[28]

Abbreviations: EBNA: Epstein–Barr nuclear antigen; EBNA-LP: EBNA leader protein; CXCL: C-X-C motif ligand; LMP: latent membrane protein; CD40: cluster of differentiation 40; IRF: interferon regulatory factor; NF-kB: nuclear factor kappa-light-chain-enhancer of activated B-cells; IL10: interleukin 10.

**Table 2:** Genomic coordinates and mature sequences of known/predicted EBV miRNAs.

miRbase Accession	miRNA	Mature Sequence ^1^	Coordinates (bp) ^2^	Refs.
MI0001064	BHRF1-1	4—UAACCUGAUCAGCCCCGGAGUU—25	41,471–41,536	[84]
MI0001065	BHRF1-2-5p BHRF1-2-3p	6—AAAUUCUGUUGCAGCAGAUAGC—27 41—UAUCUUUUGCGGCAGAAAUUGA—62	42,848–42,912	
MI0001066	BHRF1-3	3—UAACGGGAAGUGUGUAAGCACA—24	42,966–43,030	
MI0001067	BART1-5p BART1-3p	6—UCUUAGUGGAAGUGACGUGCUGUG—29 42—UAGCACCGCUAUCCACUAUGUC—63	139,346–139,415	[84,85]
MI0001068	BART2-5p BART2-3p	3—UAUUUUCUGCAUUCGCCCUUGC—24 39—AAGGAGCGAUUUGGAGAAAAUAAA—62	152,745–152,806	[84]
MI0003725	BART3-5p BART3-3p	12—ACCUAGUGUUAGUGUUGUGCU—32 49—CGCACCACUAGUCACCAGGUGU—70	139,076–139,154	[85,86]
MI0003726	BART4-5p BART4-3p	9—GACCUGAUGCUGCUGGUGUGCU—30 47—CACAUCACGUAGGCACCAGGUGU—69	139,220–139,295	
MI0003727	BART5-5p BART5-3p	15—CAAGGUGAAUAUAGCUGCCCAUCG—38 57—GUGGGCCGCUGUUCACCU—74	139,661–139,749	
MI0003728	BART6-5p BART6-3p	18—UAAGGUUGGUCCAAUCCAUAGG—39 57—CGGGGAUCGGACUAGCCUUAGA—78	140,016–140,107	
MI0003729	BART7-5p BART7-3p	15—CCUGGACCUUGACUAUGAAACA—36 51—CAUCAUAGUCCAGUGUCCAGGG—72	146,425–146,510	
MI0003730	BART8-5p BART8-3p	14—UACGGUUUCCUAGAUUGUACAG—35 49—GUCACAAUCUAUGGGGUCGUAGA—71	146,759–146,840	
MI0003731	BART9-5p BART9-3p	14—UACUGGACCCUGAAUUGGAAAC—35 52—UAACACUUCAUGGGUCCCGUAGU—74	146,946–147,032	
MI0003732	BART10-5p BART10-3p	18—GCCACCUCUUUGGUUCUGUACA—39 53—UACAUAACCAUGGAGUUGGCUGU—75	147,304–147,393	
MI0003733	BART11-5p BART11-3p	14—UCAGACAGUUUGGUGCGCUAGUUG—37 52—ACGCACACCAGGCUGACUGCC—72	147,524–147,609	
MI0003734	BART12	49—UCCUGUGGUGUUUGGUGUGGUU—70	147,888–147,970	
MI0003735	BART13-5p BART13-3p	15—AACCGGCUCGUGGCUCGUACAG—36 52—UGUAACUUGCCAGGGACGGCUGA—74	148,512–148,597	
MI0003736	BART14-5p BART14-3p	14—UACCCUACGCUGCCGAUUUACA—35 48—UAAAUGCUGCAGUAGUAGGGAU—69	148,731–148,815	
MI0004988	BART15	47—GUCAGUGGUUUUGUUUCCUUGA—68	139,507–139,584	[86]
MI0004989	BART16	20—UUAGAUAGAGUGGGUGUGUGCUCU—43	139,776–139,874	
MI0004990	BART17-5p BART17-3p	22—UAAGAGGACGCAGGCAUACAAG—43 60—UGUAUGCCUGGUGUCCCCUUAGU—82	139,894–139,995	
MI0004991	BART18-5p BART18-3p	31—UCAAGUUCGCACUUCCUAUACA—52 67—UAUCGGAAGUUUGGGCUUCGUC—88	145,932–146,050	
MI0004992	BART19-5p BART19-3p	18—ACAUUCCCCGCAAACAUGACAUG—40 57—UUUUGUUUGCUUGGGAAUGCU—77	148,198–148,290	
MI0004993	BART20-5p BART20-3p	21—UAGCAGGCAUGUCUUCAUUCC—41 56—CAUGAAGGCACAGCCUGUUACC—77	148,319–148,417	
MI0010627	BART21-5p BART21-3p	12—UCACUAGUGAAGGCAACUAAC—32 46—CUAGUUGUGCCCACUGGUGUUU—67	145,503–145,578	[83]
MI0010628	BART22	43—UUACAAAGUCAUGGUCUAGUAGU—65	147,161–147,231	

^1^ Numbers denote the start/end bases within the coordinate range that are predicted/known to produce mature miRNAs. ^2^ Coordinates mapped by original authors to prototypical wildtype genome (GenBank AJ507799.2) or B95-8 strain.
